# Mental health symptoms identify workers at risk of long-term sickness absence due to mental disorders: prospective cohort study with 2-year follow-up

**DOI:** 10.1186/s12889-015-2580-x

**Published:** 2015-12-12

**Authors:** Marieke F. A. van Hoffen, Catelijne I Joling, Martijn W. Heymans, Jos W. R. Twisk, Corné A. M. Roelen

**Affiliations:** ArboNed Occupational Health Service, Utrecht, The Netherlands; Department of Epidemiology and Biostatistics, VU University Medical Center, VU University, Amsterdam, The Netherlands; Department of Health Sciences, University Medical Center Groningen, University of Groningen, Groningen, The Netherlands; ArboNed, PO Box 11391, 3004 EJ Rotterdam, The Netherlands

**Keywords:** Absenteeism, Mass screening, Mental disorders, Mental health, Psychiatric disorders, Risk assessment, Secondary prevention, Sick leave

## Abstract

**Background:**

Mental health problems are a leading cause of long-term sickness absence (LTSA). Workers at risk of mental LTSA should preferably be identified before they report sick. The objective of this study was to examine mental health symptoms as predictors of future mental LTSA in non-sicklisted workers.

**Methods:**

Prospective cohort study of 4877 non-sicklisted postal workers. Mental health symptoms were measured at baseline in November 2010 with the Four-Dimensional Symptom Questionnaire (distress and depressed mood) and Maslach’s Burnout Inventory (fatigue). Mental health symptom scores were analyzed against incident mental LTSA retrieved from an occupational health register in 2011 and 2012. The area under the receiver operating characteristic curve (AUC) represented the ability of mental health symptom scores to discriminate between workers with and without mental LTSA during 2-year follow-up.

**Results:**

Complete cases analysis included 2782 (57 %) postal workers of whom 73 had mental LTSA during 2-year follow-up. Distress fairly (AUC = 0.75; 95 % CI 0.67–0.82) and both depressed mood (AUC = 0.64; 95 % CI 0.57–0.72) and fatigue (AUC = 0.61; 95 % CI 0.53–0.69) poorly discriminated between workers with and without mental LTSA during 2-year follow-up. The discriminative ability of distress did not improve by adding depressed mood and fatigue.

**Conclusions:**

Measurement of distress sufficed to identify non-sicklisted postal workers at risk of future mental LTSA. The Four-Dimensional Symptom Questionnaire distress scale is a promising tool to screen working populations for of mental LTSA, which enables secondary preventive strategies.

## Background

Mental disorders are the leading cause of sickness absence and disability pensions in European countries [1]. The international Labour Organization put the costs of productivity loss, disability, and unemployment due to mental disorders at 3-4 % of a country’s gross domestic product [2]. Sickness absence due to mental illness is often long-lasting [3–7]. In The Netherlands the median duration of mental sickness absence has increased from 87 days in 2005 to 118 days in 2013 [8, 9]. The probability of resuming work decreases with increasing sickness absence duration [10, 11]. In addition, prejudices and negative attitudes towards persons with mental illness may perpetuate sickness absence due to mental disorders [12, 13]. Therefore, workers with an increased risk of mental illness should preferably be identified before they report sick. For this purpose, we need tools to screen non-sicklisted workers for risk of long-term sickness absence (LTSA) due to mental disorders.

Mental health symptoms are most obvious to identify workers at risk of mental LTSA. Several studies have reported prospective associations of psychological distress [14, 15], depressed mood [16–18] and fatigue [14, 19–21] with (mental) LTSA. However, prospective associations do not tell us whether these mental health symptoms identify non-sicklisted workers with an increased risk of mental LTSA. Few studies have investigated mental health symptoms as prognostic factors for all cause LTSA [22–24]. Thorsen et al. investigated the predictive value of the 5-item Mental Health Inventory (MHI-5) for LTSA in a random sample of 4153 Danish workers [22]. A one standard deviation increase in the MHI-5 score was associated with a 37 % increase in LTSA risk. However, LTSA causes were not available so that the authors could not assess the predictive value of MHI-5 for mental LTSA. Roelen et al. [23] investigated the Four-Dimensional Symptom Questionnaire (4DSQ) as prognostic instrument to predict future mental LTSA in 1137 non-sicklisted Dutch office workers. They reported that the 4DSQ distress scale, but not the scales measuring depression, anxiety and somatization discriminated between workers who did and did not develop mental LTSA during 1-year follow-up [23]. In the same study population, fatigue was a prognostic risk factor of mental LTSA in men, but not in women [24].

The objective of the present study was to examine instruments measuring mental health symptoms (distress, depressed mood, and fatigue) as tools to predict incident mental LTSA in non-sicklisted workers. If mental health symptom scores identify workers at risk of future mental LTSA, then we could develop strategies aimed at preventing mental LTSA.

## Methods

### Study population and design

**Fig. 1:**
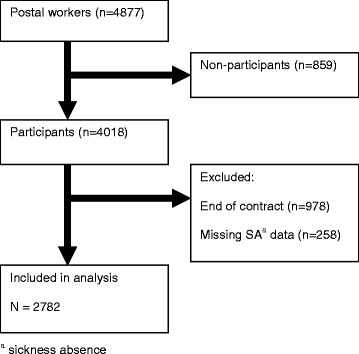
Study population flow chart

Prospective cohort study with 2-year follow-up of 4877 non-sicklisted postal workers. At baseline (November 2010), the postal workers received a questionnaire about mental health, job demands, job resources, and work ability. A total of 4018 (82 %) non-sicklisted postal workers completed the questionnaire. The questionnaire data were linked to incident mental LTSA occurring in the period January 2011 through December 2012 by citizen service number, a unique personal number assigned to every citizen registered in the Dutch Municipal Personal Records Database. Sickness absence data were not available for 1236 postal workers either because of missing or incorrect citizen service numbers (*n* = 258) or because temporary contracts ended during 2-year follow-up (*n* = 978). Consequently, 2782 (57 %) postal workers were included in complete cases analyses (Fig. 1). The Medical Ethics Committee of the University Medical Center Groningen granted ethical clearance for this study.

### Mental health symptoms

Distress was measured at baseline with the 4DSQ, which has been validated for use in the working population [25]. The 4DSQ distress scale consists of 16 items (score range 0–32; Cronbach’s α = 0.96) addressing symptoms elicited by stressors or the efforts to maintain psychosocial functioning, such as worrying, irritability, tension, listlessness, poor concentration, sleeping problems and demoralisation [25, 26]. Depressed mood was measured at baseline with four items (During the past month, did you feel that: everything is meaningless, life is not worth while, you can't enjoy anything anymore, there is no escape from your situation; α = 0.90) derived from the 4DSQ depression scale [25, 26]. Fatigue was measured at baseline with the 5-item exhaustion scale (α = 0.95) of the Dutch Maslach Burnout Inventory–General Survey [27]. For comparability, all scale scores were standardized as percentage of the maximum score (range 0–100).

### Outcome variable

Sickness absence was defined as temporary paid leave from work due to any (i.e., work-related as well as non-work-related) injury or illness and was recorded from the first to the last absence day in an occupational health register. In The Netherlands, sickness absence is employer-compensated when medically certified by an occupational physician (OP) within 42 days of reporting sick. Consequently, long-term sickness absence (LTSA) was defined as lasting ≥42 consecutive days. OPs certified sickness absence with a diagnostic code derived from the 10th International Classification of Diseases (ICD-10). LTSA certified with a diagnostic code within the ICD-10 chapter V (Mental and Behavioural Disorders) in 2011 was the outcome variable of the study. There was a gap between baseline (November 2010) and the start of follow-up (January 2011), but none of the postal workers initiated mental LTSA between November 2010 and January 2011.

### Statistical analysis

Statistical analyses were done in IBM SPSS Statistics for Windows, version 20.0 (IBM Corp. Armonk, NY, released 2011). Baseline mental health symptom scores were associated with the occurrence of mental LTSA (no = 0, yes = 1) during 2-year follow-up by logistic regression analyses. The mental health symptoms were highly intercorrelated, particularly distress and depressed mood (Pearson correlation *r* = 0.75), which corresponds with the previously reported overlap for the 4DSQ distress and depression scales [25]. To bypass collinearity, we calculated sum scores for combinations of mental health symptoms and performed logistic regression analyses with these sum scores as independent variables. Associations between mental health symptom scores and mental LTSA were checked by using spline regression curves, which revealed that all associations were linear. Furthermore, associations between mental health symptom scores and mental LTSA did not change with age and did not differ across gender and job [data not shown].

The ability of mental health symptom scores to discriminate between workers with (‘cases’) and without (‘non-cases’) incident mental LTSA during 2-year follow-up was investigated with receiver operating characteristic (ROC) analysis [28]. The area under the ROC-curve (AUC) is a measure for the discriminative ability of mental health symptom scores. If we randomly select one worker from the group of cases and one from the group of non-cases, then the AUC indicates the probability that the mental health symptom score correctly identifies a worker as case. AUC = 0.50 reflects no discrimination above chance; AUC ≥0.90 represents perfect, 0.80–0.89 good, 0.70–0.79 fair, 0.60–0.69 poor, and <0.60 failing discrimination [29].

## Results

The postal workers (*n* = 1236) whose questionnaire results could not be linked to recorded sickness absence data were younger, more often male and working as postmen with shorter job tenure than postal workers (*n* = 2782) whose questionnaire results could be linked to recorded sickness absence (Table 1). Baseline mental health symptom scores did not differ between postmen with and without sickness absence data.

**Table 1:** Population characteristics (*N* = 4018)

	Included in complete cases analyses (*n* = 2782)		Excluded because of missing SA^a^ data (*n* = 1236)		Analysis
	Median (IQR^b^)	*n* (%)	Median (IQR^b^)	*n* (%)	
Age	49.9 (9.5)		34.1 (14.9)		*P* < 0.01^c^
Gender					*P* < 0.01^d^
Men		1235 (44)		609 (49)	
Women		1547 (56)		627 (51)	
Job tenure	5.2 (1.0)		1.7 (1.1)		*P* < 0.01^e^
Work hours per week	22.4 (12.2)		10.6 (7.4)		*P* < 0.01^e^
Job					*P* < 0.01^d^
Postmen		1046 (38)		1083 (88)	
Post sorters		1455 (52)		64 (5)	
Supervisor/manager		150 (5)		8 (1)	
Other		131 (5)		81 (6)	
Mental health symptoms (range 0–100)					
Distress	25.0 (9.0–53.1)		25.0 (6.3–53.1)		*P* = 0.89^e^
Depressed mood	0.0 (0.0–25.0)		0.0 (0.0–25.0)		*P* = 0.12^e^
Fatigue	32.0 (12.0–48.0)		28.0 (8.0–48.0)		*P* = 0.29^e^

^a^ Sickness absence

^b^ Interquartile range

^c^ *t*-test for independent samples

^d^ Chi-square test

^e^ Mann–Whitney *U*-test

During 2-year follow-up, 679 postal workers had incident LTSA: 336 due to musculoskeletal disorders, 270 due to other somatic disorders (49 % non-specified illness, 12 % cardiovascular disease, 12 % respiratory disease, 11 % gastrointestinal disease, 16 % other specified illness), and 73 due to mental disorders. Postal workers with mental LTSA had higher median scores on distress (40.5, interquartile range [IQR] 12.5 – 87.5), depressed mood (25.0, IQR 0.0 – 50.0), and fatigue (40.0, IQR 20.0 – 68.0) than postal workers without mental LTSA during follow-up, scoring 25.0 (IQR 9.4 – 53.1; Mann–Whitney *P* < 0.01), 0.0 (IQR 0.0 – 25.0; Mann–Whitney *P* < 0.01), and 28.0 (IQR 8.0 – 48.0, Mann–Whitney *P* < 0.01) on distress, depressed mood, and fatigue, respectively.

**Fig. 2:**
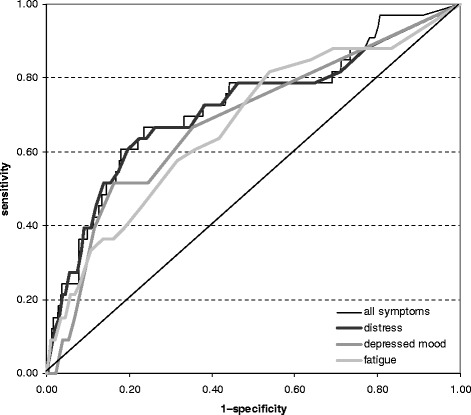
Discrimination graph. The figure shows the receiver operating characteristic (ROC) curve for different mental health symptom scores as well as the combination of all mental health scores; the diagonal indicates no discrimination above chance

Baseline mental health symptom scores were significantly associated with the occurrence of mental LTSA during 2-year follow-up (Table 2). Distress fairly discriminated between cases and non-cases and both depressed mood and fatigue poorly. The combination of distress with depressed mood and fatigue did not improve discrimination between cases and non-cases of mental LTSA during 2-year follow-up (Fig. 2). Mental health symptoms failed to discriminate between postal workers with and without LTSA due to all causes (Table 2).

**Table 2:** Mental health symptom scores and long-term (≥42 days) sickness absence (LTSA)

Mental health symptom	Score (0–100)	Association with mental LTSA	Discrimination Mental LTSA All LTSA	
	Median (IQR)^a^	OR (95 % CI)^b^	AUC (95 % CI)^c^	AUC (95 % CI)^c^
Distress	25.0 (9.0–53.1)	1.17 (1.09 – 1.27)	0.75 (0.67 – 0.82)	0.56 (0.53 – 0.58)
Depressed mood	0.0 (0.0–25.0)	1.16 (1.07 – 1.26)	0.64 (0.57 – 0.72)	0.53 (0.51 – 0.56)
Fatigue	32.0 (12.0–48.0)	1.15 (1.04 – 1.27)	0.61 (0.53 – 0.69)	0.56 (0.54 – 0.59)
Distress + depressed mood	15.6 (4.7–39.1)	1.19 (1.10 – 1.28)	0.75 (0.67 – 0.82)	0.55 (0.53 – 0.58)
Distress + fatigue	27.4 (12.5–49.6)	1.20 (1.09 – 1.32)	0.74 (0.66 – 0.81)	0.57 (0.54 – 0.59)
Depressed mood + fatigue	20.0 (8.0–36.5)	1.21 (1.10 – 1.33)	0.65 (0.57 – 0.72)	0.56 (0.54 – 0.58)
Distress + depressed mood + fatigue	20.6 (8.1–41.0)	1.22 (1.11 – 1.31)	0.75 (0.67 – 0.83)	0.56 (0.54 – 0.58)

^a^ Mean (interquartile range) standardized symptom score (range 0 to 100)

^b^ Odds ratio (95 % confidence interval) for a 10-point change in standardized mental health symptom score

^c^ Area under the receiver operating characteristic curve (95 % confidence interval)

Table 3 shows the 4DSQ distress cut-off scores with sensitivities and specificities >0.40; positive predictive values were 0.02 and negative predictive values 0.99 for all cut-off scores shown in the table.

**Table 3:** Cut-off points for the distress scale (range 0–32)

Cut-off	Number (Percent)	Sensitivity	Specificity
>6	1747 (63)	0.73	0.43
>7	1641 (59)	0.73	0.47
>8	1536 (55)	0.73	0.51
>9	1427 (51)	0.70	0.55
>10	1341 (48)	0.66	0.58
>11	1249 (45)	0.61	0.61
>12	1162 (42)	0.53	0.64
>13	1073 (39)	0.49	0.67
>14	1002 (36)	0.49	0.70
>15	918 (33)	0.48	0.72
>16	832 (30)	0.44	0.75
>17	765 (28)	0.44	0.77

## Discussion

The risk of long-term (≥42 days) sickness absence (LTSA) due to mental disorders increased with distress, depressed mood, and fatigue scores. The 4DSQ distress scale discriminated between workers who did and did not develop mental LTSA during 2-year follow-up. Combining distress with depressed mood and fatigue scores did not improve discrimination.

The current findings confirm previously reported associations between mental health symptoms and mental LTSA. Previous research has shown that the 4DSQ distress scale discriminates between office workers with and without mental LTSA during 1-year follow-up [23], which corroborates the present findings in postal workers. Depressed mood measured with a 4-item scale derived from the 4DSQ poorly discriminated postal workers with mental LTSA from those without mental LTSA, which confirms earlier findings in Dutch office workers [23]. Depressed mood may have been under-reported because of shame or anxiety of being prejudiced or stigmatized by superiors and colleagues [8, 9]. This could also explain why depressed mood scores were lower than distress and fatigue scores among postal workers. Fatigue measured with Maslach’s Burnout Inventory (MBI) poorly discriminated between postal workers with and without mental LTSA, which is also in line with previous results in a heterogeneous sample of Dutch workers [30].

Our study adds that combinations of distress with depressed mood and fatigue did not improve discrimination between workers who did and did not develop mental LTSA during 2-year follow-up. This may be due to the overlap between mental health symptoms. Terluin et al. reported that the 4DSQ distress scale shares 35-45 % of its variance with the other 4DSQ scales, particularly the depression scale [25]. The probability of depressive and anxiety disorders has been found to increase with 4DSQ distress scores [31]. Distress might not only reflect the effort an individual has put into coping with psychosocial stressors, but might also result from coping with mood and other psychiatric disorders [32].

An alternative explanation why depressed mood did not improve discrimination might be the relatively low contribution of the depressed mood scale score. Depressed mood scores were much lower than distress scores and may have had little effect on the combined (i.e., distress and depressed mood) sum score. In addition, the majority of mental LTSA episodes in the Dutch workforce is OP-certified as being due to stress-related disorders (ICD-10 F43) [33]. Although mild depressive symptoms may occur, psychological distress is most characteristic for stress-related disorders. Besides distress, fatigue is a core symptom of many mental disorders, particularly burnout and depression. Distressed workers who experience fatigue have been reported to abandon social roles, such as the work role more often than distressed workers without fatigue [25]. Our present study, however, showed that the combination of distress and fatigue did not better discriminate between postal workers with and without mental LTSA during follow-up than distress alone. From these findings, we concluded that it would be appropriate to use the 4DSQ distress scale as tool to identify workers still at work, but at risk of mental LTSA.

### Methodological considerations

The prospective design and large sample size are strengths of the study, although the study population is not likely to be representative of the Dutch workforce. Although the response rate was high (75 %), many young postmen with short job tenure and working few hours/week were excluded from the analysis. They probably had temporary seasonal contracts. Consequently, post sorters with permanent contracts were over-represented in the study population. Furthermore, workers with complete data had higher mental health symptom scores than workers whose sickness absence data could not be linked to questionnaire results. This might have over-estimated associations between mental health symptoms and mental LTSA. However, it was reassuring that the discriminative ability of the 4DSQ distress scale was of the same magnitude as previously described for Dutch office workers. In prognostic research, the generalizability of results depends on the number of settings rather than the representativeness of the study population [34].

The low number of mental LTSA episodes would restrict the statistical power of the study when we would have added covariates to the analyses. Based on preliminary analyses we concluded that there was no need to include age, gender, or job in the analyses. As all analyses only included one independent variable, there was sufficient statistical power for estimating logistic regression coefficients, even with only 73 mental LTSA events.

Another limitation of the study was potential diagnostic misclassification. LTSA due to symptoms and signs not elsewhere classified (ICD-10 chapter XVIII), such as pain, weakness and tiredness were classified as LTSA due to somatic disorders, while some of these symptoms might be indicative of mental disorders. Furthermore, LTSA had to be certified within one ICD-10 chapter and comorbidities could not be recorded on the medical certificate. We dealt with potential misclassification and comorbidities by using LTSA due to all causes as outcome. Mental health symptoms failed to discriminate between postal workers with and without LTSA due to all causes.

The present results apply to the Dutch setting where sickness absence is certified within 42 days of reporting sick. It remains to be investigated whether or not workers at risk of mental LTSA can be identified in countries where shorter sickness absence episodes are medically certified. Shorter LTSA episodes are more common in the workforce. Consequently, the statistical power to detect workers at risk of mental LTSA will increase when shorter mental LTSA episodes are included. Alternatively, shorter mental LTSA episodes are less costly and the risk of exclusion from the workforce will be lower for workers experiencing a short episode of mental LTSA compared to workers suffering a long (≥42 days) episode of mental LTSA. This raises the question of the practical relevance in terms of the benefits and harm of screening for risk of short mental LTSA episodes. It is debatable whether it is beneficial to identify workers for interventions to prevent short mental LTSA episodes if this would lead to unnecessary stigmatization and increased utilization of health services.

### Practical implications

The 16-item 4DSQ distress scale fairly discriminated between cases and non-cases of mental LTSA during 2-year follow-up. An AUC = 0.75 indicates that if we randomly draw a worker from the group with mental LTSA and a workers from the group without mental LTSA, the distress scale will assign the highest risk of mental LTSA to the worker from the group with mental LTSA in 75 % of the cases. If the current findings are confirmed in other working populations, then the 4DSQ distress scale could be used as tool to identify non-sicklisted workers with an increased risk of mental LTSA. Workers with high distress scores could then be referred for further mental health assessment and preventive treatment if appropriate.

The choice of a distress cut-off score depends on the objectives of case finding. Given the fact that mental LTSA has a median duration of 118 days [9] and the average productivity costs in The Netherlands are €30 ($35; £23) per hour [35], the median costs of mental LTSA episode may amount to more than €25,000 ($29,000; £19,000) for a full-time worker. These costs would plead for low distress cut-off scores so that as much cases as possible are identified and treated to prevent mental LTSA. We should be careful, however, not to medicalize transient psychological distress. Furthermore, mental LTSA is a rare event and, therefore screening for mental LTSA with low distress cut-off scores will identify more false-positives than true positives (i.e., mental LTSA cases). Thus, healthcare providers might choose distress cut-off scores with high specificity to only identify the highest risk workers and restrict false-positives. All the more because interventions aimed at reducing LTSA were found to be cost-effective among workers with a high LTSA risk, but not among workers with a moderately increased LTSA risk [36].

Apart from identifying individual workers for preventive interventions, the results of screening for risk of mental LTSA could be accumulated at the organizational level. Preventive interventions can then be targeted at departments or settings where many workers are at risk of mental LTSA. A review of intervention programs showed that combinations of person-directed and organization-directed interventions was most effective to prevent burnout, one of the main mental LTSA diagnoses [37].

## Conclusions

The 4DSQ distress scale is a promising tool to identify non-sicklisted workers at risk of mental LTSA, which provides opportunities for developing strategies to prevent mental LTSA. The addition of other mental health symptoms, such as depressed mood and fatigue did not improve risk discrimination. The 4DSQ distress scale should be further investigated as tool to screen working populations for risk mental LTSA.

## Abbreviations

4DSQ: Four-Dimensional Symptom Questionnaire
AUC: Area under the receiver operating characteristic
ICD-10: 10th International Classification of Diseases
LTSA: Long-term (i.e., ≥42 days) sickness absence
MBI: Maslach Burnout Inventory

## References

[1] Organization for Economic Cooperation and Development (2012). Sick on the Job? Myths and Realities about Mental Health and Work.

[2] Scheil-Adlung X, Sandner L (2010). Paid sick leave: incidence, patterns and expenditure in times of crisis. ESS Paper N^o^ 27.

[3] Foss L, Gravseth HM, Kristensen P, Claussen B, Mehlum IS, Skyberg K (2010). Risk factors for long-term sickness absence due to mental sickness: a register-based 5-year follow-up from the Oslo Health Study. J Occup Environ Med.

[4] Knudsen AK, Øverland S, Aakvaag HF, Harvey SB, Hotopf M, Mykletun A (2010). Common mental disorders and disability pension award: seven year follow-up of the HUSK study. J Psychosom Res.

[5] Stansfeld SA, Fuhrer R, Head I (2011). Impact of common mental health disorders on sickness absence in an occupational cohort study. Occup Environm Med.

[6] Ahola K, Virtanen M, Honkonen T, Isometsä E, Aromaa A, Lönnqvist J (2011). Common mental disorders and subsequent disability: a population-based Health 2000 Study. J Affect Disord.

[7] Knudsen AK, Harvey SB, Mykletun A, Øverland S (2013). Common mental disorders and long-term sickness absence in a general working population. The Hordaland Study. Acta Psychiatr Scand.

[8] Roelen CA, Koopmans PC, Hoedeman R, Bültmann U, Groothoff JW, van der Klink JJ (2009). Trends in the incidence of sickness absence due to common mental disorders between 2001 and 2007 in the Netherlands. Eur J Public Health..

[9] National Institute for Public Health and the Environment. http://www.nationaalkompas.nl/gezondheid-en-ziekte/functioneren-en-kwaliteit-van-leven/ziekteverzuim-en-arbeidsongeschiktheid/wat-is-de-relatie-met-ziekten-en-aandoeningen/ Accessed 23 May 2015.

[10] Labriola M (2008). Conceptual framework of sickness absence and return to work, focusing on both the individual and contextual level. Work.

[11] Lund T, Kivimäki M, Labriola M, Villadsen E, Christensen KB (2008). Using administrative sickness absence data as a marker of future disability pension: the prospective DREAM study of Danish private sector employees. Occup Environ Med..

[12] Baumann AE (2007). Stigmatization, social distance and exclusion because of mental illness: the individual with mental illness as a ‘stranger’. Int Rev Psychiatry.

[13] Knapstad M, Øverland S, Henderson M, Holmgren K, Hensing G (2014). Shame among long-term sickness absentees: correlates and impact of subsequent sickness absence. Scand J Public Health.

[14] Bültmann U, Huibers MJH, van Amelsvoort LG, Kant I, Kasl SV, Swaen GM (2005). Psychological distress, fatigue and long-term sickness absence: prospective results from the Maastricht Cohort Study. J Occup Envrion Med..

[15] Terluin B, van Rhenen W, Anema JR, Taris TW (2011). Psychological symptoms and subsequent sickness absence. Int Arch Occup Environ Health..

[16] Bültmann U, Rugulies R, Lund T, Christensen KB, Labriola M, Burr H (2006). Depressive symptoms and the risk of long-term sickness absence: a prospective study among 4747 Employees in Denmark. Soc Psychiatry Epidemiol.

[17] Lexis MA, Jansen NW, van Amelsvoort LG, van den Brandt PA, Kant I (2009). Depressive symptoms as a predictor of sickness absence among the working population. J Occup Environ Med.

[18] Hjarsbech PU, Andersen RV, Christensen KB, Aust B, Borg V, Rugulies R (2011). Clinical and non-clinical depressive symptoms and risk of long-term sickness absence among female employees in the Danish eldercare sector. J Affect Disord.

[19] Janssen N, Kant IJ, Swaen GMH, Janssen PP, Schröer CA (2003). Fatigue as a predictor of sickness absence: results from the Maastricht Cohort Study on fatigue at work. Occup Environ Med..

[20] Bültmann U, Nielsen MB, Madsen IEH, Burr H, Rugulies R (2013). Sleep disturbances and fatigue: independent predictors of sickness absence? A prospective study among 6538 employees. Eur J Public Health..

[21] Roelen CA, van Rhenen W, Groothoff JW, van der Klink JJL, Bültmann U (2014). Prolonged fatigue is associated with sickness absence in men but not in women: prospective study with 1-year follow-up of white collar employees. Int Arch Occup Environ Health..

[22] Thorsen SV, Rugulies R, Hjarsbech PU, Bjørner JB (2013). The predictive value of mental health for long-term sickness absence: the Major Depression Inventory (MDI) and the Mental Health Inventory (MHI) compared. BMC Med Res Methodol..

[23] Roelen CAM, Hoedeman R, van Rhenen W, Groothoff JW, van der Klink JJ, Bültmann U (2013). Mental health symptoms as prognostic risk markers of all-cause and psychiatric sickness absence in office workers. Eur J Public Health.

[24] Roelen CAM, Heymans MW, van Rhenen W, Groothoff JW, Twisk JW, Bültmann U (2014). Fatigue as prognostic risk marker of mental sickness absence in white collar employees. J Occup Rehabil..

[25] Terluin B, van Rhenen W, Schaufeli WB, de Haan M (2004). The Four-Dimensional Symptom Questionnaire (4DSQ): measuring distress and other mental health problems in a working population. Work Stress.

[26] Terluin B, van Marwijk HW, Adèr HJ, de Vet HC, Penninx BW, Hermens MW (2006). The Four-dimensional Symptom Questionnaire (4 DSQ): a validation study of a multidimensional self-report questionnaire to assess distress, depression, anxiety and somatization. BMC Psychiatry.

[27] Bakker AB, Demerouti E, Schaufeli WB (2002). Validation of the Maslach burnout inventory – general survey: an internet study. Anx Stress Coping.

[28] Steyerberg EW, Vickers AJ, Cook NR, Gerds T, Gonen M, Obuchowski M (2010). Assessing the performance of prediction models: a framework for traditional and novel measures. Epidemiol.

[29] Fad J, Upadhye S, Worster A (2006). Understanding receiver operating characteristic (ROC) curves. Can J Emerg Med..

[30] Roelen CA, van Hoffen MF, Groothoff JW, de Bruin J, Schaufeli WB, van Rhenen W. Can the Maslach Burnout Inventory and Utrecht Work Engagement Scale be used to screen for risk of long-term sickness absence? Int Arch Occup Environ Health. 2015. doi10.1007/s00420-014-0981-2 10.1007/s00420-014-0981-2 25212752

[31] Terluin B, Brouwers EPM, van Marwijk HWJ, Verhaak PFM, van der Horst HE (2009). Detecting depressive and anxiety disorders in distressed patients: comparative diagnostic accuracy of the Four-Dimensional Symptom Questionnaire (4DSQ) and the Hospital Anxiety and Depression Scale (HADS). BMC Fam Pract..

[32] Clark LA, Watson D (1991). Tripartite model of anxiety and depression: psychometric evidence and toxonomic implications. J Abnorm Psychol..

[33] Roelen CA, Norder G, Koopmans PC, van Rhenen W, van der Klink JJ, Bültmann U (2012). Employees sick-listed with mental disorders: who returns to work and when?. J Occup Rehabil..

[34] Justice AC, Covinsky KE, Berlin JA (1999). Assessing the generalizability of prognostic information. Ann Intern Med..

[35] Tan SS, Bouwmans CAM, Rutten FFH, Hakkaart-van Roijen L (2012). Update of the Dutch manual for costing in economic evaluations. Int J Technol Asses Health Care..

[36] Taimela S, Justén S, Aronen P, Sintonen H, Läärä E, Malmivaara A (2008). An occupational health intervention programme for workers at high risk for sickness absence. Cost effectiveness analysis based on a randomised controlled trial. Occup Environ Med.

[37] Awa WL, Plaumann M, Walter U (2010). Burnout prevention: a review of intervention programs. Patient Educ Couns..

